# The influence of neighbourhood green space on children’s physical activity and screen time: findings from the longitudinal study of Australian children

**DOI:** 10.1186/s12966-015-0288-z

**Published:** 2015-09-30

**Authors:** Taren Sanders, Xiaoqi Feng, Paul P. Fahey, Chris Lonsdale, Thomas Astell-Burt

**Affiliations:** School of Science and Health, Western Sydney University, Parramatta, 2150 NSW Australia; Early Start Research Institute, University of Wollongong, Wollongong, 2722 NSW Australia; Menzies Centre for Health Policy, University of Sydney, Sydney, 2006 NSW Australia; School of Health and Society, University of Wollongong, Wollongong, 2722 NSW Australia; Institute for Positive Psychology and Education, Australian Catholic University, Sydney, 2135 NSW Australia; School of Geography and Geosciences, University of St Andrews, St Andrews, KY16 9AL UK

**Keywords:** Green space, Physical activity, Screen time, Children, Longitudinal data

## Abstract

**Objective:**

It is often hypothesised that neighbourhood green space may help prevent well-known declines in physical activity and increases in sedentary behaviour that occur across childhood. As most studies in this regard are cross-sectional, the purpose of our study was to use longitudinal data to examine whether green space promotes active lifestyles as children grow older.

**Methods:**

Data came from participants (*n* = 4983; age = 4–5) of the Longitudinal Study of Australian Children, a nationally representative study on health and child development. Physical activity and screen time were measured biennially (2004–2012) using questionnaires and time use diaries. Quantity of neighbourhood green space was objectively measured using Australian Bureau of Statistics mesh block data for each participant’s statistical area level 2. Multilevel regression was used to test for associations between physical activity and screen time with green space quantity, adjusting for socio-economic confounders.

**Results:**

Boys living in areas with 10 % more neighbourhood green space had a: 7 % (95 % CI = 1.02, 1.13) greater odds of choosing physically active pastimes; 8 % (95 % CI = 0.85, 1.00) lower odds of not enjoying physical activity; 2.3 min reduction in weekend television viewing (95 % CI = −4.00, −0.69); and 7 % (95 % CI = 1.02; 1.12) and 9 % (95 % CI = 1.03; 1.15) greater odds of meeting physical activity guidelines on weekdays and weekends, respectively. No statistically (or practically) significant results were observed for girls.

**Conclusion:**

Current provisions of neighbourhood green space may be more amenable to promoting active lifestyles among boys than girls. Research is needed to explore what types of green space promote active lifestyles in all children.

## Introduction

An increasing number of studies have reported on the utility of neighbourhood green space as a promoter of physically active lifestyles [1–3], though evidence has not been unequivocal [4, 5]. Differences in the benefit of green space by age and gender may be among many possible reasons for these inconsistent results [6], though few studies have sought to investigate in such detail, especially with longitudinal data [7]. In youth populations, for example, cross-sectional studies have tended to show positive associations between neighbourhood green space and physical activity [8–10]. A recent longitudinal study of Australian children aged 6–13 years, however, noted that a higher level of neighbourhood green space was associated with lower body mass index (BMI) for boys, but not for girls [11]. Moreover, that association was not consistent across childhood, but emerged as boys grew older. The results from the Australian study may indicate that the relationship between green space and health does not extend to all individuals equally. It is therefore probable that any relationships between green space and physical activity would also be contingent upon age and gender, in line with what is known about differences in autonomy that manifest between boys and girls of different ages [12]. Evidence of this nature is required to highlight if current provisions of green space promotes health and active lifestyles among all children, or just particular age groups and genders. Accordingly, the purpose of this study was to examine the patterning of physical activity and sedentary behaviour among girls and boys between the ages of 4 and 13 in relation to neighbourhood green space, and to investigate whether any such patterns varied as children grew older.

## Methods

### Data

Data for this study came from the older cohort of the Longitudinal Study of Australian Children (LSAC), a large-scale government project run by the Australian Department of Families, Housing, Community Services, and Indigenous Affairs. Full details of the LSAC methodology are published elsewhere [13]. In brief, a two-stage clustered design was used, with eligible children identified through Australia’s universal healthcare database, Medicare. Children were considered eligible if they were born between March 1999 and February 2000. The postcodes in which these children lived were then stratified by state, and then by urban or rural status. A random sample of 1-in-10 postcodes were then chosen with the children residing within those postcodes comprising the sample. A total of 9893 children were approached to participate by mail-out letter. Of those approached, 50.4 % were successfully recruited, with 37.5 % choosing to opt-out and 15.2 % unable to be contacted. Excluding those who were unable to be contacted, the overall response rate was 59.4 % [13]. Data collection commenced in 2004, when the children were aged 4–5years old. Data were collected from the same children every 2 years, primarily by face-to-face interviews with the children’s parents, with additional data coming from the child’s other caregivers (e.g., teachers), census-linked data, and the children. The Australian Institute of Family Studies Ethics Committee provided ethics approval for the LSAC, and all participants provided written informed consent.

### Green space

To estimate residential green space, land-use data was extracted from the Australian Bureau of Statistics (ABS) mesh blocks from 2006 [14]. Mesh blocks are used to classify very small land parcels according to their main land use. We isolated all mesh blocks that were classified as ‘parkland’ from other forms of land-use, including ‘farmland’ which would not typically be publically accessible. Neighbourhood green space was derived based on the child’s statistical area level 2 (SA2) value; the smallest area unit available in the LSAC [15]. Generally, each SA2 has a residential population ranging between 3000 and 25,000 individuals and was designed by the Australian Bureau of Statistics (ABS) to be representative of communities [15]. In the current study, approximately 1200 SA2s were measured. The mean population of the SA2s in which the study participants resided was approximately 11,000 (standard deviation ~ 6000). The neighbourhood green space measure for each participant was the proportion of total land surface designated as green space in their SA2 of residence. A similar methodology has demonstrated association between this measure of green space and health outcomes among adults [1, 16, 17] and children [11]. As a 1 % difference in green space was unlikely to have practical relevance, the green space measure was rescaled by dividing by 10, making a unit change the equivalent to a 10 % difference in green space.

### Physical activity and sedentary behaviour

Time spent physically active or sedentary was measured using direct and indirect measures. Time use diaries (TUDs) were used to directly assess children’s behaviour over a short measurement period at each time point. Questionnaires were administered to the parents of the children at each data collection point to measure perceptions of physical activity and screen time. Due to limitations in the data, not all measures were available at every time point. Information on when each measure was available, as well as sample sizes, is provided in Table 1.

**Table 1:** Sample characteristics

	Wave 1		Wave 2		Wave 3		Wave 4		Wave 5	
	Boys	Girls	Boys	Girls	Boys	Girls	Boys	Girls	Boys	Girls
Sample, n (%)	2537 (50.9 %)	2446 (49.1 %)	2277 (51.0 %)	2187 (49.0 %)	2212 (51.1 %)	2119 (48.9 %)	2133 (51.2 %)	2036 (48.8 %)	2021 (51.1 %)	1935 (48.9 %)
Age, Mean (SD)	4.2 (0.4)	4.2 (0.4)	6.3 (0.5)	6.3 (0.5)	8.3 (0.4)	8.3 (0.4)	10.3 (0.5)	10.3 (0.5)	12.4 (0.5)	12.4 (0.5)
Green Space, Mean % (SD)	19.0 (16.5)	19.1 (16.4)	19.0 (16.8)	19.1 (16.2)	19.4 (16.9)	19.1 (16.5)	19.4 (16.9)	19.0 (16.7)	19.5 (17.0)	19.2 (16.8)
Weekly Family Income (In Thousands), Mean $ (SD)	1.5 (2.6)	1.6 (2.4)	1.8 (1.2)	1.8 (1.2)	2.1 (1.5)	2.1 (1.5)	2.2 (2.0)	2.3 (2.1)	2.6 (2.0)	2.5 (1.7)
Maternal Education, Mean Years (SD)	14.4 (2.6)	14.4 (2.7)	14.6 (2.5)	14.6 (2.7)	14.8 (2.5)	14.7 (2.6)	14.9 (2.5)	14.9 (2.6)	15.0 (2.5)	15.0 (2.6)
Child Indigenous Status, n (%)	91 (3.6 %)	96 (3.9 %)	74 (3.3 %)	79 (3.6 %)	59 (2.7 %)	65 (3.1 %)	56 (2.6 %)	62 (3.1 %)	57 (2.8 %)	56 (2.9 %)
Child Speaks Language Other Than English, n (%)	318 (12.5 %)	306 (12.5 %)	268 (11.8 %)	249 (11.4 %)	246 (11.1 %)	220 (10.4 %)	224 (10.5 %)	218 (10.7 %)	168 (8.3)	159 (8.2 %)
Impartial or Does Not Enjoy Physical Activity, n (%)	169 (6.7 %)	165 (6.8 %)	.	.	.	.	132 (6.3 %)	146 (7.3 %)	160 (8.2 %)	227 (12.0 %)
Chooses active activities during free time, n (%)	845 (33.4 %)	600 (24.6 %)	838 (36.8 %)	399 (18.3 %)	698 (31.6 %)	488 (23.1 %)	.	.	429 (21.5 %)	233 (12.2 %)
Weekday TV, Mean Minutes (SD)	.	.	99.0 (66.2)	94.7 (63.7)	103.8 (78.7)	99.1 (74.4)	111.6 (77.0)	105.4 (73.6)	122.2 (90.5)	118.5 (84.3)
Weekend TV, Mean Minutes (SD)	.	.	148.0 (89.4)	140.98 (83.8)	158.5 (93.7)	156.2 (90.4)	175.5 (95.7)	178.0 (92.6)	188.5 (99.9)	192.4 (97.9)
TUD Weekday Physical Activity, Mean Minutes (SD)	66.1 (77.0)	59.1 (72.1)	83.0 (81.5)	74.2 (83.6)	84.7 (91.0)	76.0 (90.4)	142.3 (102.2)	125.9 (95.6)	116.8 (98.9)	84.9 (88.7)
Meets Physical Activity Guidelines Weekday, n (%)	684 (41.6 %)	597 (38.0 %)	833 (58.8 %)	655 (47.4 %)	696 (54.3 %)	587 (47.1 %)	976 (79.3 %)	881 (73.0 %)	901 (67.3 %)	675 (50.6 %)
TUD Weekend Physical Activity, Mean Minutes (SD)	98.4 (100.8)	91.1 (94.9)	173.8 (121.6)	147.9 (115.6)	159.4 (129.4)	137.0 (122.6)	144.6 (115.0)	130.1 (117.7)	136.7 (120.4)	98.7 (105.6)
Meets Physical Activity Guidelines Weekend, n (%)	925 (57.2 %)	815 (54.9 %)	1286 (82.0 %)	1097 (77.8 %)	1028 (77.1 %)	868 (69.9 %)	255 (73.1 %)	188 (66.0 %)	240 (68.8 %)	180 (55.4 %)
TUD – Weekday Screen Time, Mean Minutes (SD)	66.1 (77.0)	59.1 (72.1)	83.0 (81.5)	74.2 (83.6)	84.7 (91.0)	76.0 (90.4)	142.3 (102.2)	125.9 (95.6)	116.8 (98.9)	84.9 (88.7)
Meets Screen Time Guidelines Weekday, n (%)	816 (49.3 %)	925 (58.0 %)	1090 (70.1 %)	1142 (78.3 %)	818 (61.5 %)	883 (68.3 %)	439 (35.2 %)	564 (46.4 %)	509 (37.8 %)	513 (37.9 %)
TUD – Weekend Screen Time, Mean Minutes (SD)	98.4 (100.8)	91.1 (94.9)	173.8 (121.6)	147.9 (115.6)	159.4 (129.4)	137.0 (122.6)	144.6 (115.0)	130.1 (117.7)	136.7 (120.4)	98.7 (105.6)
Meets Screen Time Guidelines Weekend, n (%)	674 (41.6 %)	747 (49.7 %)	590 (38.7 %)	673 (47.9 %)	394 (29.6 %)	456 (36.5 %)	48 (13.6 %)	61 (21.4 %)	103 (29.2 %)	86 (25.5 %)

Note: *n* number of participants, *SD* standard deviation, *%* proportion of participants with data, · data not available at waves

#### Choice of free time

Children’s parents were asked two questions from the Amherst questionnaire [18] regarding their child’s activity habits. To measure choice of free time activities, parents were asked “*What does* [child] *usually do when she/he has a choice about how to spend free time?*” and asked to choose from three options that most represented their child. Parents who chose “*Usually chooses active pastimes*” were coded as active, while parents who chose “*Usually chooses inactive pastimes*” or “*Just as likely to choose active as inactive pastimes*” were coded as inactive or impartial. This question has previously been reported to have a test-retest reliability of 0.88 [18]. Children who voluntarily choose to spend free time participating in active activities have been found to have an overall higher level of physical activity [19].

#### Physical activity enjoyment

To measure physical activity enjoyment, parents were asked “*How much does* [child] *enjoy physical activity or exercise?*”, with responses given on a 5-point Likert scale (1 = very much dislikes activity; 5 = very much likes activity). This question has previously been reported to have a test-retest reliability of 0.87 [18]. While enjoyment of physical activity is a known predictor of physical activity for both male and female children [20, 21], it may not reflect actual participation in physical activity. Therefore, enjoyment was included to test if children in greener areas enjoy physical activity more than those in low green space areas do. For the purposes of analysis, this variable was recoded as a binary outcome. However, as the dislikes activity option was much less frequently chosen than the likes activity option, coding was reversed [22]. As such, 1–3 was coded as 1 (is impartial to, or does not enjoy, physical activity) and 4–5 was coded as 0 (enjoys physical activity).

#### Television viewing

Parents were asked to estimate the amount of time children spent watching television (TV) on a typical weekday and a typical weekend day (i.e., “*About how many minutes on a typical weekday, would you say that* [child] *watches TV or videos at home?”*)*.* Answers were recorded as whole minutes, and entered as a continuous variable into the models.

#### Time use diaries (TUDs)

Two types of TUDs were used to measure children’s health behaviours. For the first three time points (i.e., waves 1–3), a “light” time use diary was completed by one of the child’s primary caregiver (usually the biological mother) over two 24-h periods (one weekday, one weekend day) [23]. The respondent completed the diary by colouring bubbles that corresponded with activities the child participated in during 96 15-min periods. The respondent chose from a list of 26 pre-coded activities, and could select up to six activities for each period to allow for concurrent activities (i.e., watching television while eating).

For the last two time points (i.e., waves 4–5), a TUD was administered to the child. Rather than choosing from pre-coded activities, children were asked to record the sequence of activities over the course of a single randomly allocated day. An interviewer then input the information recorded by the child, as well as additional contextual information, during an interview with the child on the day following the diary completion. A coding framework was used to code the children’s activities [24], so as to make diaries comparable across children.

To process the TUD data, the total amount of time in activities representing physical activity or screen time were calculated. For example, “*walk for travel or for fun*” was coded as physical activity, while “*watching TV, video, DVD, movie*” was coded as screen time. Variables for weekday and weekend minutes physical activity and minutes screen time were then generated. To account for the reduced opportunity for physical activity and screen time during school hours, a dummy variable to represent if the child attended school was included for the weekday estimates. As previous research has found differences in the relationship between public open spaces and weekday and weekend physical activity [9], we chose not to combine these measures. To measure the influence of green space on meeting health guidelines, the TUD estimates were also transformed into binary variables, based on whether or not the child met the Australian guidelines for levels of physical activity (>60 min/day) and screen time (<120 min/day) [25]. Due to the different respondents and TUD types, we deemed it inappropriate to include both types of TUDs in the same model. Therefore, waves 1–3 and waves 4–5 were modelled separately.

### Socioeconomic circumstances

Recent Australian evidence suggests that socioeconomic circumstances may predict physical activity habits of children [26]. Further, ethnicity has proven to be a consistent predictor of physical activity levels [26, 27]. Therefore, in order to address possible confounding, measures of family socioeconomic circumstances and ethnicity were included. Specifically, combined weekly income of caregivers (in thousands) and the number of years of education the mother had received [28] were included as socioeconomic indicators. If the child spoke a language other than English (LOTE) at home, and if the child was of Australian Aboriginal or Torres Strait Islander heritage [29] were included as ethnicity indicators.

### Statistical analysis

Descriptive statistics of all measures were used to determine the characteristics of the sample. Multilevel linear regression and multilevel logistic models were then used to test the association between green space and each measure of physical activity or screen time. Within each model, the children’s health behaviour at each survey wave (level 1) was nested within individuals (level 2) to examine longitudinal associations [30]. Children were also grouped by their SA2 (level 3) in order to model spatial clustering.

**Fig. 1:**
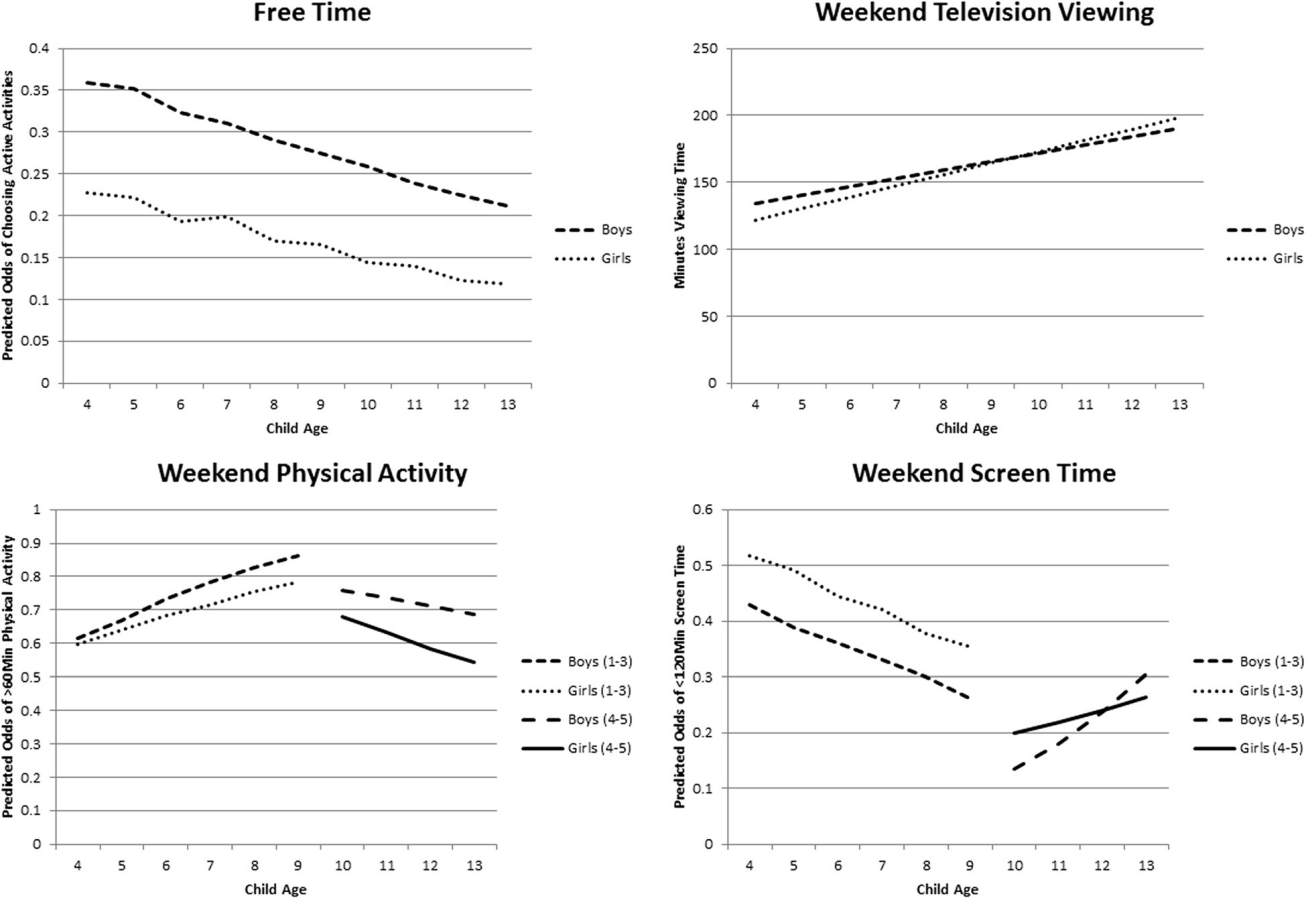
Unadjusted, gender-stratified mean trajectories of child physical activity and screen time behaviour over time

To examine the role of child gender on the outcome variables, unadjusted mean trajectories were fit with an age-by-gender interaction. Examples of the results are provided in Fig. 1. Significant gender differences in the mean trajectories were seen for all outcomes except TV viewing time. We therefore chose to fit gender stratified models for all outcomes.

For each outcome measure, an unadjusted model consisting of age, green space, and the outcome measure was fit. Confounding variables were then added successively, with log-likelihood tests computed after each model to determine whether or not each variable improved the fit. To test for variation in the effect of green space across time, a green space by age interaction was then added. As multilevel modelling is resilient to data missing at random [30], children were included where their data was available. Significance levels were set at 5 % for all tests. All statistical analyses were conducted using Stata 12 (StataCorp, College Station, TX, USA).

## Results

The results of the descriptive statistics are presented in Table 1. The vast majority of parents indicated that their children enjoyed physical activity (>90 % for both boys and girls) at younger ages. The proportion of boys who enjoyed physical activity remained stable at older ages (93.3 % at wave 1; 91.8 % at wave 5), but decreased for girls (93.2 % at wave 1; 88.0 % at wave 5). Oddly, the proportion of children who chose active activities was much smaller than those who enjoyed physical activity. This proportion appeared to decrease as children aged (33.4 to 21.5 % for boys; 24.6 to 12.2 % for girls). The number of children meeting physical activity guidelines appeared to peak at wave 4 when the children were 10–11 years old, although it is unclear if this is the result of the change in measurement methodology. It is for this reason that we chose to fit separate models for waves 1–3 and waves 4–5 for all variables derived from the TUDs.

### Choice of free time

The outcome of the multilevel models is presented in Table 2. After controlling for possible confounders, boys in areas with 10 % more green space had an average 7 % greater odds of choosing physically active pastimes (OR = 1.07, 95 % CI = 1.02, 1.13; *p* = 0.009). For girls, this relationship was not statistically significant (OR = 1.01, 95 % CI = 0.96, 1.07; *p =* 0.825). A green space by age term was not significant when added for boys or girls (*p* > 0.05).

**Table 2:** Influence of green space on children’s physical activity and screen time

	Boys				Girls			
	Unadjusted	*P*-Value	Adjusted	*P*-Value	Unadjusted	*P*-Value	Adjusted	*P*-Value
Chooses Active Free Time, OR (95 % CI)	1.06 (1.01, 1.12)	0.120	**1.07 (1.02, 1.13)**	**0.009**	1.01 (0.96, 1.07)	0.592	1.01 (0.95, 1.07)	0.805
Physical Activity Non-Enjoyment, OR (95 % CI)	0.95 (0.89, 1.02)	0.173	**0.92 (0.85, 1.00)**	**0.043**	0.94 (0.88, 1.01)	0.103	0.95 (0.87, 1.03)	0.210
Television Weekday (Minutes), β (95 % CI)	-1.04 (-2.45, 0.38)	0.150	-0.32 (-1.76, 1.11)	0.659	**-1.95 (-3.33, -0.56)**	**0.006**	-1.18 (-2.50, 0.17)	0.087
Television Weekend (Minutes), β (95 % CI)	**-2.35 (-3.97, -0.72)**	**0.005**	**-2.34 (-4.00, -0.69)**	**0.005**	-1.40 (-3.13, 0.32)	0.111	-0.16 (-1.93, 1.61)	0.858
Minutes Physical Activity (Weekday; Waves 1–3), β (95 % CI)	1.35 (-0.14, 2.83)	0.075	**1.88 (0.23, 3.53)**	**0.026**	0.56 (-1.09, 2.21)	0.506	-0.06 (-1.90, 1.77)	0.947
Minutes Physical Activity (Weekday; Waves 4–5), β (95 % CI)	-0.22 (-2.56, 2.11)	0.852	0.69 (-2.00, 3.38)	0.616	-0.65 (-2.90, 1.60)	0.572	-0.26 (-2.88, 2.35)	0.844
Minutes Physical Activity (Weekend; Waves 1–3), β (95 % CI)	**3.20 (0.82, 5.58)**	**0.008**	**3.01 (0.37, 5.66)**	**0.026**	1.47 (-0.73, 3.66)	0.190	1.18 (-1.21, 3.58)	0.333
Minutes Physical Activity (Weekend; Waves 4–5), β (95 % CI)	1.91 (-3.44, 7.26)	0.485	2.24 (-3.90, 8.38)	0.475	-1.52 (-6.36, 3.33)	0.539	-1.78 (-7.36, 3.80)	0.533
Minutes Screen Time (Weekday; Waves 1–3), β (95 % CI)	**-2.02 (-3.97, -0.07)**	**0.043**	-1.90 (-3.97, 0.17)	0.071	-0.77 (-2.63, 1.08)	0.414	-1.13 (-3.02, 0.77)	0.243
Minutes Screen Time (Weekday; Waves 4–5), β (95 % CI)	-1.38 (-4.69, 1.93)	0.412	1.22 (-2.15, 4.59)	0.478	-1.64 (-4.91, 1.63)	0.325	1.86 (-1.48, 5.19)	0.275
Minutes Screen Time (Weekend; Waves 1–3), β (95 % CI)	**-2.98 (-5.27, -0.70)**	**0.011**	-2.21 (-4.66, 0.23)	0.076	-1.97 (-4.10, 0.17)	0.071	-1.65 (-3.84, 0.54)	0.140
Minutes Screen Time (Weekend; Waves 4–5), β (95 % CI)	-5.15 (-11.96, 1.66)	0.138	-5.97 (-13.73, 1.79)	0.131	2.99 (-3.50, 9.48)	0.367	5.80 (-1.47, 13.08)	0.118
>60 min Physical Activity (Weekday; Waves 1–3), OR (95 % CI)	**1.05 (1.01, 1.10)**	**0.022**	**1.07 (1.02, 1.12)**	**0.009**	1.03 (0.98, 1.07)	0.289	1.01 (0.96, 1.06)	0.736
>60 min Physical Activity (Weekday; Waves 4–5), OR (95 % CI)	0.99 (0.94, 1.05)	0.740	1.00 (0.93, 1.07)	0.928	0.96 (0.91, 1.02)	0.211	0.98 (0.92, 1.05)	0.647
>60 min Physical Activity (Weekend; Waves 1–3), OR (95 % CI)	**1.08 (1.03, 1.14)**	**0.002**	**1.09 (1.03, 1.15)**	**0.002**	1.00 (0.96, 1.05)	0.923	0.99 (0.94, 1.04)	0.784
>60 min Physical Activity (Weekend; Waves 4–5), OR (95 % CI)	1.05 (0.94, 1.17)	0.408	1.04 (0.92, 1.18)	0.509	1.03 (0.94, 1.13)	0.467	1.04 (0.92, 1.17)	0.559
<120 min Screen Time (Weekday; Waves 1–3), OR (95 % CI)	1.03 (0.98, 1.09)	0.197	1.03 (0.97, 1.10)	0.300	1.04 (0.98, 1.10)	0.207	1.06 (0.99, 1.14)	0.073
<120 min Screen Time (Weekday; Waves 4–5), OR (95 % CI)	1.00 (0.95, 1.06)	0.991	0.98 (0.91, 1.05)	0.550	1.04 (0.98, 1.10)	0.236	0.98 (0.91, 1.05)	0.538
<120 min Screen Time (Weekend; Waves 1–3), OR (95 % CI)	**1.06 (1.01, 1.12)**	**0.032**	1.05 (0.99, 1.11)	0.126	1.04 (0.99, 1.10)	0.139	1.03 (0.97, 1.09)	0.321
<120 min Screen Time (Weekend; Waves 4–5), OR (95 % CI)	0.98 (0.86, 1.11)	0.729	0.96 (0.83, 1.11)	0.580	0.94 (0.84, 1.05)	0.285	0.98 (0.84, 1.15)	0.844

Note: Adjusted models are adjusted for child age, maternal education, if the child speaks a language other than English, family weekly income, and child indigenous status. Time use diary (weekday) is further adjusted to account for school days. Bold text indicates p<0.05

*CI* confidence intervals, *OR* odds ratio

### Physical activity enjoyment

For physical activity enjoyment, those living in areas with 10 % more green space were associated with 8 % lower odds of a boy not enjoying physical activity (OR = 0.92, 95 % CI = 0.85, 1.00; *p* = 0.043) in the adjusted models. Interestingly, the influence of green space was not significant before controlling for confounding, suggesting that green space may be susceptible to negative confounding. The trend was similar for girls, but failed to reach significance (OR: 0.95; 95 % CI = 0.87, 1.03; *p* = 0.207). No statistically significant interaction between green space and age was seen for boys or girls (*p* > 0.05).

### Television viewing

Parent reported weekday TV viewing was not significantly related to green space for boys or girls after adjustment for socio-economic circumstances (all *p* > 0.05). However, a 10 % difference in green space was associated with an adjusted mean of 2.4 min less weekend TV viewing time for boys (β = 2.34, 95 % CI = −4.00, −0.69; *p* = 0.005), but not girls (β = −0.16, 95 % CI = −1.93, 1.61; *p* = 0.858). When a green space × age interaction was fit, green space was more strongly related with television viewing as girls grew older (βGreen Space = −5.76, 95 % CI = −10.53, −0.98; *p* = 0.018; βGreen Space*Age = 0.62, 95 % CI = 0.13, 1.11; *p* = 0.014). The interaction effect was not present for boys (*p* > 0.05).

### Time use diaries

#### Physical activity (Minutes/Day)

After adjusting for other predictors, green space did not significantly predict girls’ time in physical activity on weekdays or weekends during waves 1–3 or waves 4–5. However, for boys a 10 % difference in green space was associated with a mean of 1.9 min greater time spent physically active on a weekday (β = 1.88, 95 % CI = 0.22, 3.53; *p* = 0.026), and 3.0 min more weekend physical activity (β = 3.01, 95 % CI = 0.37, 5.66; *p* = 0.026) after adjusting for confounders, but only at younger ages (i.e., waves 1–3). For waves 4–5, a green space by age interaction indicated green space became less strongly related with physical activity as children grew older (β_Green Space_ = 69.59, 95 % CI = 6.11, 133.06; *p* = 0.032; β_Green Space*Age_ = −5.92, 95 % CI = −11.47, 0.37; *p* = 0.037).

#### Screen time (Minutes/Day)

Living in an area with 10 % more green space was associated with a mean of 2.0 min less weekday (β = −2.02, 95 % CI = −3.97, −0.07; *p* = 0.043) and 3.0 min less weekend (β = −2.98, 95 % CI = −5.27, −0.70; *p* = 0.011) screen time for boys at younger ages (i.e., waves 1–3). However, the effect was reduced to 1.9 less weekday minutes (β = −1.90, 95 % CI = −3.97, 0.17; *p* = 0.071) and 2.2 less weekend minutes (β = 2.21, 95 % CI = −4.66, 0.23; *p* = 0.076) and rendered non-significant after adjusting for confounders. While the individual effect was not significant for girls, an interaction effect between green space and age was present, such that green space became more strongly related with screen time as girls grew older (β_Green Space_ = −7.11, 95 % CI = −12.79, −1.42; *p* = 0.014; β_Green Space*Age_ = 0.98, 95 % CI = 0.10, 1.86; *p* = 0.029). There were no associations for boys or girls at older ages (i.e., waves 4–5).

#### Adherence to physical activity and screen time guidelines

The odds of a girl meeting physical activity recommendations (i.e., >60 min) on a weekday were not significantly influenced by green space availability at waves 1–3 or waves 4–5. For boys, a 10 % difference in green space was associated with greater odds of meeting physical activity guidelines on both weekdays (OR = 1.07, 95 % CI = 1.02, 1.12; *p* = 0.009) and weekends (OR = 1.09, 95 % CI = 1.03, 1.15; *p* = 0.002), but only at waves 1–3. When a green space by age interaction term was added, a statistically significant interaction was found for boys at waves 1–3, such that green space became less strongly related to adherence to physical activity guidelines as boys aged (OR_Green Space_ = 2.12, 95 % CI = 1.01, 4.44; *p* = 0.046; OR_Green Space*Age_ = 0.94, 95 % CI = 0.88, 1.00; *p* = 0.043). Boys’ weekend physical activity was significantly associated with green space, with 10 % more green space associated with 9 % greater odds of meeting physical activity guidelines (OR = 1.09, 95 % CI = 1.03, 1.15; *p* = 0.047), at waves 1–3. There were no statistically significant associations between green space and the odds of either boys or girls meeting the screen time recommendations, on weekdays or weekends.

## Discussion

The objective of this study was to examine the longitudinal relationship between neighbourhood green space and children’s physical activity and screen time behaviours. Overall, neighbourhood green space was associated with more physical activity for boys, although effect sizes were modest. Similarly, green space was associated with a reduction in boys’ weekend screen time and parent-reported TV viewing time. Further, green space was associated with greater odds of a boy enjoying physical activity, and choosing physical active past times. However, influences on physical activity and screen time were only seen when the children were at a younger age. Therefore, the key finding of this longitudinal study is that boys’ physical activity and screen time are impacted by their neighbourhood green space quantity.

To contextualise the findings, a boy living in an area with 10 % green space would do an average of 108 h less physical activity between the ages of 4 and 13, compared to a boy living in an area with 20 % green space. Averaged across the 9 years, there is a loss of approximately 14 min per week of physical activity. The difference further increases when compared to a boy living in an area with 50 % green space, with the child in the low green space area doing approximately 55 min per week less on average than the child in the higher green space area. The effect is the opposite for screen time – a boy living in a 10 % green space area would have an average of 12 min per week more screen time compared to a boy in a 20 % green space area, and 48 min more compared to a boy in a 50 % green space area. 

It is noteworthy that primary green space effects were only observed for boys. In previous research on the same sample of children, only boys’ BMI was significantly influenced by levels of neighbourhood green space [11]. As increased physical activity and decreased screen time are often touted as potential causal mechanisms for green space related health gain, it is not surprising that the trend is similar for both outcomes. In research on adult populations, positive health effects of green space were found almost exclusively for men, with little or no significant influences on women’s health [31]. It is possible that even with equal availability of green space, girls would be less likely to use the space than boys. It has been demonstrated that males significantly outnumber females in public parks (a major component of green space) in the USA, and that males are almost twice as likely as females to engage in vigorous physical activity while there [32]. In research on children, results are often not analysed separately for boys and girls, making comparisons to previous research difficult and possibly masking significant sex-related differences (where sex by green space interactions are not specified). It is unclear if gender differences seen are a result of differences in green space quality or facilities, or if the gender differences manifest as a result of differing levels of autonomy between boys and girls [33]. Identifying ways to make green space more appealing to girls may be an important avenue in developing green spaces that maximize population health benefit.

Among the strengths of the present study is the use of an objective measure of green space and the use of longitudinal data from a nationally representative sample of Australian children. The use of longitudinal data analysis to investigate the relationship between green space and health or health behaviours is regularly recommended [34], but difficult to accomplish. Further, we employed multiple measures of physical activity and screen time, from both the parent and the child.

The potential limitations of this study should also be noted. As with all longitudinal studies, participant dropout may produce unintended bias. Sample weights are provided for the LSAC data; however, we chose not to apply them as our interests lay in the associations with green space, rather than estimating prevalence. Additionally, as this was not a randomized controlled trial, there is always a risk of unmeasured confounders influencing the results. For example, the influence of green space on physical activity or screen time may be explained by socioeconomic confounding beyond what we adjusted for, such as value of assets. Further, while our measures of physical activity and screen time included objective measures, study design choices outside of our control were not ideal. For example, the change of measurement tool for the TUDs between waves 3 and 4 resulted in a less-than-optimal modelling strategy. While we attempt to minimize this shortcoming with multiple measurement methods, it is acknowledged that a more consistent physical activity or screen time measurement tool may yield different results.

While the measure of green space measure was objective, the land use of ABS mesh blocks were categorised at only one time point. We have not corrected for any variations in the amount of land designated as parkland over the duration of the study. Furthermore, the measure of green space only quantified the amount of green space in an area, and could not account for type or quality of green space. It is likely that green space quality plays an important role in green space’s relationships with physical activity and screen time [35]. In particular, green space quality may explain the different results for boys and girls. However, data on green space or park quality are not routinely collected. Future research should continue to investigate the longitudinal influence of green space on physical activity or screen time. In particular, there is a need for studies using reliable, objective measures of physical activity and screen time, as well as of green space. A closer investigation into the reason for apparent gender differences in green space influence on physical activity and screen time is also warranted.

## Conclusions

Neighbourhood green space was associated with greater chance of boys choosing physically active activities, and enjoying physical activity. More green space was also associated with greater odds of boys meeting physical activity recommendations on weekends, and with less weekend TV viewing time. These results indicate that neighbourhood green space may promote more active lifestyles among young boys. Therefore, urban planners and policy makers should be mindful that inadequate access to green space may further compound the growing problem of youth inactivity.
